# Interaction of Lysozyme with Sulfated β-Cyclodextrin: Dissecting Salt and Hydration Contributions

**DOI:** 10.3390/molecules31020372

**Published:** 2026-01-20

**Authors:** Jacek J. Walkowiak

**Affiliations:** 1 DWI—Leibniz-Institute for Interactive Materials e.V, Forckenbeckstraße 50, 52074 Aachen, Germany; jacek.walkowiak@uni-bayreuth.de; 2 Institute of Technical and Macromolecular Chemistry, RWTH Aachen University, Worringerweg 2, 52074 Aachen, Germany; 3 Aachen-Maastricht Institute for Biobased Materials (AMIBM), Maastricht University, Urmonderbaan 22, 6167 RD Geleen, The Netherlands; 4 Department of Chemistry, Inorganic Chemistry III, Northern Bavarian NMR Centre, University of Bayreuth, Universitätsstrasse 30, 95440 Bayreuth, Germany

**Keywords:** thermodynamic analysis, isothermal titration calorimetry (ITC), hydration, protein, cyclodextrin

## Abstract

This article investigates the thermodynamic driving force of the interaction between lysozyme (Lys) and sulfated β-cyclodextrin (β-CDS), with a particular emphasis on the elusive role of hydration during polyelectrolyte–protein binding. Using isothermal titration calorimetry (ITC), the binding affinity was quantified across varying temperatures and salt concentrations, employing a recently developed thermodynamic framework that explicitly separates the contributions from counterion release and hydration effects. The study reveals that while counterion release is minimal in the Lys/β-CDS system, hydration effects become a dominant factor influencing the binding free energy Δ*G*_b_, especially as experimental temperature deviates from the characteristic temperature *T*_0_. It demonstrates that hydration contributions can substantially weaken binding at increased salt concentration *c*_s_. The high characteristic temperature *T*_0_ and the salt-dependent heat capacity change indicate a complex interplay of water structure and ion association—significantly departing from commonly linear interpretations of Δ*G*_b_ vs. *log c*_s_ based solely on counterion release effects. This work advances the understanding of polyelectrolyte–protein interactions by providing the first direct quantification of the hydration effect in such complexes and may have an impact on the rational design of biomolecular assemblies and therapeutic carriers.

## 1. Introduction

The interaction between polyelectrolytes and proteins is a cornerstone of molecular biophysics, with charged biopolymers such as DNA and heparin playing fundamental roles in numerous biological processes [1,2,3]. Understanding these interactions is essential, as they govern key events ranging from DNA–protein binding to the formation of therapeutic complexes [4,5,6]. Decades of research have established that electrostatic attraction is often the principal driving force in these systems, particularly through the release of condensed counterions from highly charged polyelectrolytes upon protein binding [7,8,9,10,11]. This counterion release results in a favorable entropic contribution to the free energy of complex formation.

However, the critical role of water in these interactions is less straightforward yet equally important. The formation of polyelectrolyte–protein complexes often involve not only the displacement of counterions but also the uptake or release of water molecules [12,13,14,15]. These subtle hydration effects can significantly influence the free energy of binding, especially at elevated salt concentrations [16,17], and are closely linked to phenomena such as enthalpy–entropy compensation—long considered a hallmark of both biological and synthetic systems [18,19,20,21,22]. While the thermodynamic consequences of counterion release have been thoroughly characterized, the interplay among salt, water activity, and binding energetics remains an area of active investigation.

Among the diverse proteins investigated for drug design and therapeutic applications, lysozyme (Lys) holds particular significance [23]. Lysozyme is a globular protein with a molecular weight of 14.3 kDa, consisting of 129 amino acid residues—including 11 anionic and 19 cationic residues—and stabilized by four intramolecular disulfide bridges [24,25,26]. Its surface is predominantly polar, with hydrophobic amino acids largely sequestered in the interior, resulting in a structure optimized for solubility and interaction with polarized partners [26,27]. Lysozyme is widely distributed in nature and found in a variety of cells [25]. Functionally, lysozyme is renowned for its antibacterial activity, specifically targeting the peptidoglycan layer of Gram-positive bacterial cell walls by catalyzing the hydrolysis of glycosidic bonds, leading to bacterial lysis [28]. Owing to this property, lysozyme has broad utility as an antimicrobial agent. Moreover, it exhibits anticancer, antiviral, and anti-inflammatory activities, which expand its potential for biomedical applications [29,30]. Notably, studies have demonstrated that lysozyme can reversibly bind various endogenous and exogenous ligands, positioning it as a promising drug carrier [25,31,32]. These unique biochemical and structural characteristics make lysozyme an exemplary model protein for probing molecular mechanisms in drug metabolism, transport, and polyelectrolyte–protein interactions. Hen egg white lysozyme was selected for this study due to its availability, high purity, and acceptance as a standard reference protein in biophysical and pharmaceutical research. Its structure, folding, and reaction to binding partners are extensively characterized, facilitating direct mechanistic comparison with prior polyelectrolyte–protein work and establishing a strong basis for future application-driven research involving human lysozyme [23,24,25,26].

Alongside proteins such as lysozyme, cyclodextrins (CDs) and their derivatives are playing an increasingly important role in pharmaceutical science. Cyclodextrins are cyclic oligosaccharides, typically comprising six to eight α-D-glucopyranose units linked via α-(1,4) glycosidic bonds, and exhibit a truncated cone-shaped structure [33]. This architecture features a hydrophobic central cavity surrounded by a hydrophilic outer surface, imparting cyclodextrins with amphiphilic properties that facilitate host–guest chemistry [34]. Through a characteristic “fit-and-find” recognition mechanism, CDs can form stable inclusion complexes with a wide variety of guests [35]. This ability substantially enhances the solubility and bioavailability of poorly water-soluble drugs, thus improving their therapeutic performance and enabling efficient passage across biological membranes [34,35,36]. Moreover, native cyclodextrins can self-assemble in aqueous solutions to form aggregates, where the aggregate characteristics are dictated by their solubility and concentration [37]. The combination of inclusion complex formation and self-assembly provides cyclodextrins with significant versatility as pharmaceutical excipients, supporting diverse applications in controlled drug delivery and formulation design. Especially noteworthy are sulfated cyclodextrins, which emerge when cyclodextrins are chemically modified with sulfate groups [38,39,40]. These derivatives retain structural adaptability and biocompatibility while acquiring new biological functions; notably, they have recently gained recognition as promising heparin substitutes owing to their ability to mimic the behavior of glycosaminoglycans (GAGs) [41]. Sulfated cyclodextrins demonstrate potential for specific binding to P- and L-selectins [42], making them valuable candidates for targeted drug delivery.

Recent advances—especially those involving dendritic polyglycerol sulfate (dPGS) and glycosaminoglycans (GAGs) like heparin—have shown that isothermal titration calorimetry (ITC) is a powerful tool for precisely quantifying the binding interactions between polyelectrolytes and proteins [2,3,11,21,43]. ITC not only measures the strength of binding (free energy) but also reveals important details about the mechanisms involved, such as the release of counterions and changes in hydration that occur during binding [44]. Despite these capabilities, it remains challenging to directly observe and quantify the hydration effects accompanying polyelectrolyte–protein interactions. Thus, by studying Lys—a well-characterized protein model with well established, broad bioactivity [25,28,29,30,31,32], and β-CDS—an increasingly relevant biocompatible polyanion and drug delivery agent [34,35,36,38,39,40]—this work aims to fully disentangle the contributions of counterion release and hydration in polyelectrolyte–protein binding. To achieve this, a recently developed thermodynamic framework [15] is employed that quantitatively incorporates both counterion and water effects to better explain binding energetics in these systems and provide insights that may be applicable in the future design of advanced protein–polymer hybrid systems for pharmaceutical uses.

## 2. Theory

The theory of complex formation between polyelectrolytes and proteins has been detailed in earlier works [3,15,44,45]. Nevertheless, a summary of the key equations used for evaluating the binding free energy Δ*G*_b_ is necessary. The binding constant *K*_b_, typically determined with high accuracy by isothermal titration calorimetry [2,20,21,22,43,46] is directly related to Δ*G*_b_(*T, c*_s_) through the following:

(1) $$∆G_{b}\left(T,\;c_{s}\right)=\;-RTlnK_{b}$$

Δ*G*_b_ can be evaluated in a model-free way using the generalized van’t Hoff expression:

(2) $$∆G_{b}=∆H_{b,ref}-T∆S_{b,ref}+∆C_{p}\left[T-T_{ref}-Tln(\frac{T}{T_{ref}})\right]$$

where Δ*H*_b,ref_ and Δ*S*_b,ref_ denote the enthalpy and the entropy of binding at a reference temperature *T*_ref_ that can be chosen freely [47,48]. This equation allows analysis of Δ*G*_b_(*T*) for a given salt concentration *c_s_* and does not account for simultaneous *T* and *c_s_* variation. Moreover, since the specific heat capacity Δ*C*_p_ is related to the second derivative of Δ*G*_b_ with respect to *T*, its experimental determination is challenging, and only approximate values can be obtained. To address these issues, a modified expression has been developed [15], describing Δ*G*_b_ as a function of both *T* and *c*_s_:

(3) $$∆G_{b}\left(T,c_{s}\right)=RT∆n_{ci}lnc_{s}+∆H_{0}-T∆S_{0}+c_{s}\frac{d∆C_{p}}{dc_{s}}\left[T-T_{o}-Tln\left(\frac{T}{T_{0}}\right)\right]$$

The first term in Equation (3) accounts for counterion release quantified by Δ*n*_ci_, the latter being the net number of ions released during binding. The last term, with a characteristic temperature *T*_0_ and Δ*C*_p_ scaling with *c*_s_ via the $\frac{d∆C_{p}}{dc_{s}}$ coefficient, represents the hydration effect on Δ*G*_b._ Equation (3) can be re-written with a residual free energy of binding Δ*G*_res_ *=* Δ*H*_0_ − *T*_0_Δ*S*_0_, comprising all contributions to Δ*G*_b_ other than the ones derived from counterion release and hydration effects, as follows:

(4) $$∆G_{b}\left(T,c_{s}\right)=RT∆n_{ci}lnc_{s}-RT0.036∆wc_{s}+∆G_{res}$$

Which introduces parameter Δ*w* as the effect of hydration which in turn can be derived as follows:

(5) $$∆w=\frac{\frac{d∆C_{p}}{dc_{s}}}{0.036R}\left(ln\frac{T}{T_{0}}+\frac{T_{0}}{T}-1\right)$$

Equation (5) implies that hydration effects vary with salt concentration, and the hydration contribution to Δ*G*_b_(Δ*w*) reaches an extremum at *T = T*_0_. If Δ*w* is small (i.e., when *T* is close to *T*_0_), counterion release dominates binding free energy, portraited by linear Δ*G*_b_(*T, c*_s_) vs. *log c*_s_ plots. Because hydration contributions vary quadratically with (*T − T*_0_), they become relevant only when the experimental temperature deviates significantly from *T*_0_ [45]. This explains why experimental plots of *ln K*_b_ or Δ*G*_b_ vs. *log c*_s_ are often linear, and why quantifying hydration effects is difficult to address [6,10].

## 3. Results

Interaction between β-CDS and Lys resulted in formation of a 1:1 complex characterized with a high binding constant *K*_b_ as determined by ITC. At the highest salt concentrations and the highest temperatures, a second distinct binding site for β-CDS to Lys was identified, resulting in the formation of a 1:5 complex. Table S1 gathers all binding parameters (*N*, *K*_b_ and Δ*H*_ITC_) measured directly in the ITC experiments. As the measured heat Δ*H*_ITC_ is accompanied by the linked equilibria such as the buffer ionization and thus does not represent the reaction heat alone, its dependence on *T* and *c*_s_ will not be discussed further [49,50].

**Figure 1:**
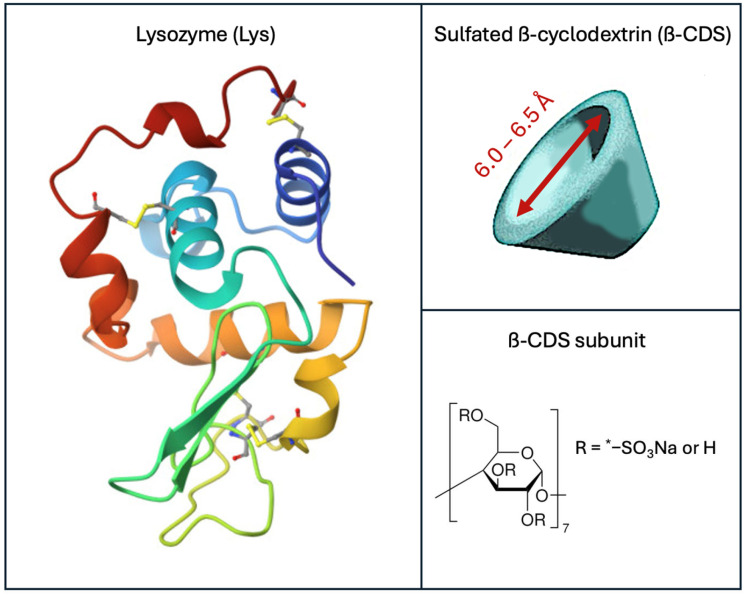
Structure of hen egg white lysozyme from the protein data bank (PDB—1DPX) and a schematic representation of a sulfated β-cyclodextrin molecule (with an emphasis on the hydrophobic central cavity) along with the chemical structure of its subunit.

As two variables (*T* and *c*_s_) are the decisive parameters that determine Δ*G*_b_, their contribution needs to be revealed separately. The binding free energies Δ*G*_b_ as a function of *T* are shown in Figure 1. Almost no dependence, within the limit of error, and a strong decrease in Δ*G*_b_ with increasing *c*_s_ is a feature observed in previous studies on the complex formation between polyelectrolytes and protein [3,10,21,51].

**Figure 2:**
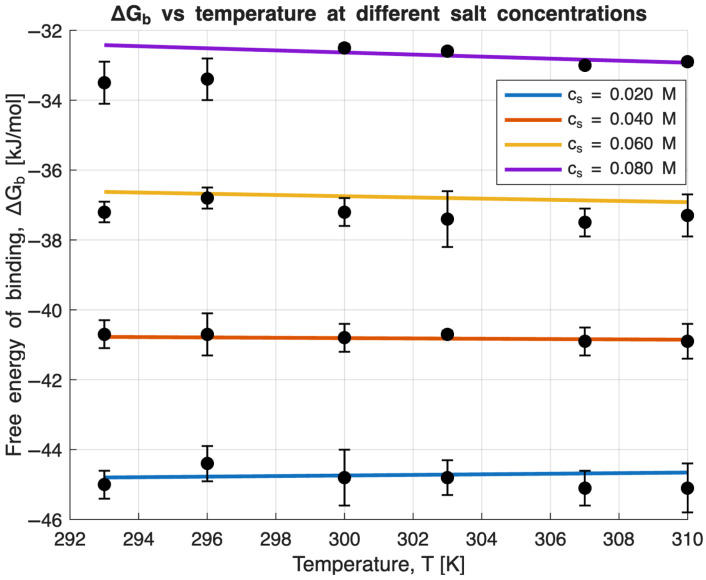
Dependence of the Gibbs free energy of binding (Δ*G*_b_) on temperature for all ionic strengths. Solid lines represent the fits obtained from Equation (3) [2]. Error bars for the *c*_s_ = 0.080 M at temperatures greater than 300 K are not visible due to their small magnitude.

**Figure 3:**
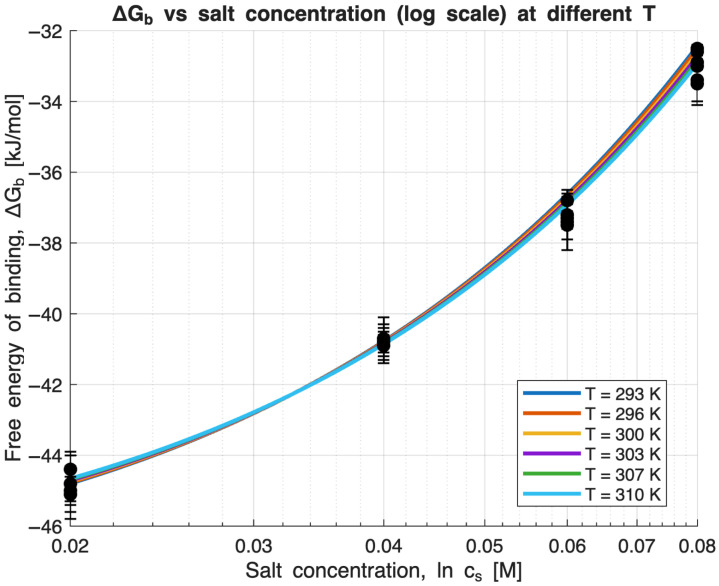
Dependence of the Gibbs free energy of binding (Δ*G*_b_) on salt concentration *c*_s_ for all temperatures. Solid lines represent the fits obtained from Equation (3) [2].

As previously outlined, Equation (3) enables a comprehensive description of the entire data set presented in Figure 1 through a single mathematical expression. The solid lines in Figure 2 and Figure 3 illustrate the optimal fits obtained using Equation (3). Altogether, the model relies on five parameters systematically adjusted to achieve the most accurate representation of the experimental data. This optimization is carried out using the MatLab script detailed in the Supplementary Material. The parameter *T*_0_ is incrementally varied to identify the fit with the lowest sum of squared errors. Notably, Δ*n*_ci_ is nearly zero (see Table 1) and is observed to remain relatively constant as *T*_0_ is changed, highlighting that the counterion release contribution to Δ*G*_b_ is negligible. Figure 2 and Figure 3 clearly demonstrate that this approach allows the entire data set to be fitted remarkably well with just a single set of parameter values. Therefore, fitting Equation (3) offers the significant advantage of utilizing all available data simultaneously, rather than only fitting subsets. One key result of this analysis is that the characteristic temperature *T*_0_ is found to be unexpectedly high with a value of 1214 K, thus differing considerably from the experimental temperatures (293–310 K). It must be noted that *T*_0_ represents the characteristic temperature at which term Δ*w,* related to the free energy of hydration, vanishes [15]. Consequently, hydration effects become observable only when the experimental temperature deviates substantially from *T*_0_, enabling quantitative analysis [3]. In this context, *T*_0_ provides a measure of the extent to which the analyzed interaction is dominated by hydration—a concept recently demonstrated to apply to protein unfolding [52]. The coefficient $\frac{d∆C_{p}}{dc_{s}}=\;-0.4\;\mathrm{kJ}/(K\;\mathrm{mol}\;M)$ shows that the specific heat capacity Δ*C*_p_ is not a constant for a given Lys/β-CDS complex but depends, to a relatively small extent, on *c*_s_. This fact is one of the central conclusions of the used model [15].

Figure 3 displays Δ*G*_b_ as a function of the logarithm of the *c*_s_, showing a clear non-linear dependence. Thus, the quantity Δ*w* in Equation (4) cannot be neglected. As Δ*w* depends quadratically on the distance *T* − *T*_0_ to the characteristic temperature [45], it comes into play only if the distance is large enough—which is the case in the present study. The resulting data are therefore suitable for analyzing the influence of hydration, as captured by the parameter Δ*w* and negligible contribution from the counterion release.

Table 2 provides all Δ*w* values, obtained from the application of Equation (5), as a function of temperature. In all instances, a large negative Δ*w* is observed, with the absolute value decreasing as the temperature increases. According to Equation (4), such negative values of Δ*w* led to a reduction in the magnitude of Δ*G*_b_ as the salt concentration *c*_s_ increases.

## 4. Discussion

It is important to recognize that the effect described by Δ*w* encompasses the hydration phenomena of both the Lys and the β-CDS. As a result, the parameters compiled in Table 1 reflect the combined hydration effects from both reaction partners, which complicate straightforward interpretation. However, based on the extensive amount of literature, certain assumptions can be made. First of all, following the discussion by Malicka et al. [3] on the solute partitioning model, [53] it can be stated that the partition coefficients *K_p_*_±_ *= m^loc^*_±_*/m^bulk^*_±_, where *m^loc^*_±_ and *m^bulk^*_±_ denote the molalities of the cations/anions in the hydrate and the bulk water phase, respectively, must be larger than unity. This is because only in this scenario will the Δ*w* parameter be negative, as approximated by the following relation $∆w≅\frac{1}{2}\left(K_{p+}+K_{p-}-2\right)∆B_{H_{2}O}$, where Δ*B*_H_2_0_ is a negative number representing the water released upon complex formation. Until now, such values of |Δ*B*_H_2_0_| ≈ 2000 were typically associated with large systems like DNA/Klentaq [54,55]. 

Furthermore, it is well-known that the formation of Lys/CDs complexes is primarily driven by interactions between the hydrophobic regions of the protein and the hydrophobic cavity of cyclodextrin [56,57]. This association frequently alters the protein structure, causing partial unfolding and a decrease in thermal stability as CDs exhibit a preference for binding to the unfolded state of proteins when a greater number of amino acids become accessible to the solvent and thus capable of interaction [23,33,36,58].

For Lys specifically, the total solvent-accessible surface area (SASA) of the folded state is calculated at 65.7 nm, with 34.94 nm accounted for by exposed nonpolar residues, 17.90 nm by exposed polar residues, and 12.86 nm by exposed charged residues. Upon unfolding, the total SASA increases to 225 nm, and previously buried amino acids become solvent-exposed and can participate in complexation with CDs [59]. Virtually all studies on Lys indicate that aromatic amino acid residues, especially tryptophan, play a central role in interaction with CDs [23,60]. In Lys, two tryptophan residues are adjacent, two are separated by two other residues, and two are isolated [61]. The accessibility of these residues—whether hidden in the hydrophobic core or exposed to the solvent—varies with the protein conformational state.

It is also evident that the energetics of Lys/CDs complex formation is a sum of multiple factors: the endothermic disruption of hydrogen bonds between water molecules inside the cavity, the exothermic formation of the hydration shell surrounding the complex, and contributions (either exothermic or endothermic) from interactions between CD and the guest aminoacidic residue. For chemically sulphated CDs like β-CDS, additional energetic factors are the hydration of the sulphate groups and their interaction with exposed charged residues. During the binding process, the CD cavity, which contains bound crystal water, competes with the solvent to access the protein binding site [59]. This leads to dehydration of the cavity, enabling the formation of an inclusion complex with aminoacidic residues. Entropically, the release of water from both the cavity and the ordered hydrophobic hydration shells of the protein into the bulk solvent must be the most significant contributor. The negative value of Δ*w* indicates that water molecules in the hydration shell of the unfolded protein have higher activity than those in bulk water. Unfolding thus requires transporting water from this lower-activity bulk state to the higher-activity shell, which demands free energy input [52].

Thus, the pronounced hydration effect identified for the Lys/β-CDS complex formation may result from an interplay of electrostatic interaction between sulphate groups of β-CDS and exposed charged residues of Lys, leading to partial unfolding of the protein and its further stabilization by the formation of an inclusion complex between the β-CDS cavity and hydrophobic aminoacidic residue of Lys. It is obvious that an in-depth analysis of the Lys/β-CDS complex formation requires further studies over Lys unfolding upon interaction with β-CDS. This claim is further amplified by the presence of the second binding site for β-CDS to Lys at high salt concentration and high temperatures (see Figure S1t,x). Analysis of the overlapping ITC titration curves suggest that rapid formation of 1:5 complex is followed by the analyzed formation of 1:1 complex. Interestingly, such ITC profiles were recorded for Lys binding with heparin [3] and phytate [62]. Since the latter shows an example of binding at low ionic strength (<20 mM), it implies that it might be a protein specific effect.

Finally, the applied model allows to obtain the parameters Δ*H*_0_ = −55 kJ/mol, Δ*S*_0_ = −0.014 kJ/(K mol), and Δ*G*_res_ = −38 kJ/mol (see Table 1). Since the latter quantity is the measure for the interaction between the polyelectrolyte and the protein in direct contact, it should be interpreted as the indication of the binding affinity and can be directly compared with other systems [3]. What is worth noting is that this quantity is more negative than the measured Δ*G*_b_ at *c*_s_ of 60 and 80 mM, showing clearly the extent to which the hydration effect weakens the Lys/β-CDS binding at higher ionic strengths. However, on the virtue of Equation (4), with a negative Δ*w* parameter, Δ*G*_res_ should be more negative than Δ*G*_b_ for all investigated *c_s_*—which is not the case for *c*_s_ of 20 and 40 mM. After performing a simple internal cross-check for *c*_s_ of 20 mM, it becomes clear that value of −38 kJ/mol must be within the lower limit of error for Δ*G*_res_, which is no greater than 20%. Such uncertainty is completely acceptable for the used model and do not hinder the overall analysis.

## 5. Materials and Methods

### 5.1. Materials

β-Cyclodextrin, sulfated sodium salt (β-CDS, ≥ 97%, degree of sulfation: 12–15 mol per mol β-CD), lysozyme from chicken egg white (Lys, lyophilized powder, protein ≥90%, ≥40,000 units/mg protein), sodium phosphate monobasic (NaHPO_4_, ≥98%), sodium phosphate dibasic (Na_2_HPO_4_, 99.95%), and sodium chloride (NaCl, ≥99%) were purchased from Merck (Darmstadt, Germany) and used directly.

### 5.2. Isothermal Titration Calorimetry

ITC experiments were conducted on a PEAQ-ITC instrument (Microcal, Northampton, MA, USA). All samples were prepared in a phosphate buffer. A total of 3.8 mM Na_2_HPO_4_ and 1.2 mM NaH_2_PO_4_ were dissolved in Milli-Q water. To adjust the ionic strength, NaCl was added individually into the buffer samples. Following this, 39 μL of Lys−buffer solution was titrated into the sample cell with 39 successive injections. Stirring rate was set to 750 rpm with a time interval of 180 or 240 s between each injection. A total of 200 μL of β-CDS solution, in a matching buffer, was contained in the sample cell. The measurements were performed at the following ionic strengths: 20, 40, 60, 80 mM and temperatures: 293, 296, 300, 303, 307, 310 K (see Table S1). Before each experiment (binding and dilution), all samples were degassed and thermostated for several minutes at 1° below the experimental temperature. Prior to the analysis of the ITC data, the heat of the protein dilution was subtracted from the corresponding heat of the Lys/β-CDS interaction. The data were evaluated with either the single set of identical binding sites (SSIS) or the two sets of independent sites (TSIS) model, where the latter assumes the presence of two different binding sites for β-CDS to Lys (see Evaluation of ITC Data in Supplementary Materials) [63,64].

## 6. Conclusions

In summary, this work presents a detailed thermodynamic examination of lysozyme binding to sulfated β-cyclodextrin, rigorously distinguishing the roles of counterion release and hydration in polyelectrolyte–protein interaction. Isothermal titration calorimetry combined with a recently developed theoretical framework revealed that counterion release has only a minor influence on the free energy of binding for the Lys/β-CDS system, while hydration effects play a prominent and previously unquantified role. The model’s application demonstrates that hydration significantly reduces the magnitude of the binding free energy at higher salt concentrations. The high characteristic temperature differing considerably from the experimental temperature emphasizes the centrality of hydration phenomena, pointing to a sophisticated interplay between water structure, ion association, and polyelectrolyte–protein interaction. These findings deepen the understanding of Lys/β-CDS interactions and provide thermodynamic data that may contribute to the design of protein–polymer functional excipients by highlighting the potential to tune binding affinities through precise modulation of hydration and ionic conditions.

## Figures and Tables

**Table 1:** Constants characterizing the β-CDS interaction with Lys ^1^.

T_0_ (K)	Δn_ci_	$\frac{d∆C_{p}}{dc_{s}}$ (kJ/(K mol M)	ΔH_0_ (kJ/mol)	ΔS_0_ (kJ/mol K)	ΔG_res_ (kJ/mol)
1214	−0.2	−0.4	−55	−0.014	−38

^1^ *T*_0_: characteristic temperature (Equation (3)); Δ*n*_ci_: net number of released counterions (Equation (3)); $\frac{d∆C_{p}}{dc_{s}}$: coefficient characterizing the dependence of Δ*w* on temperature and salt concentration (Equations (4) and (5)); Δ*H*_0_ and Δ*S*_0_: enthalpic and entropic contributions, respectively, at *T *=* T*_0_ (Equation (3)).

**Table 2:** Parameter Δ*w* as determined by application of Equation (5).

Temp. (K)	Δw ^1^
293	−2301
296	−2259
300	−2203
303	−2163
307	−2111
310	−2073

^1^ error on Δw is within the limit of 20% for all experimental temperatures.

## Data Availability

The data presented in this study are available within the article and Supplementary Materials.

## Supplementary Materials

The following supporting information can be downloaded at https://www.mdpi.com/article/10.3390/molecules31020372/s1, Table S1: Binding parameters for the Lys/β-CDS complex formation as determined by ITC; Figure S1: ITC data; Evaluation of ITC Data; Matlab script used to fit binding parameters to Equation (3) [65,66].

## Abbreviations

The following abbreviations are used in this manuscript:

Lys	Lysozyme
β-CDS	Sulfated β-Cyclodextrin
CDs	Cyclodextrins
ITC	Isothermal titration calorimetry
GAGs	Glycosaminoglycans
dPGS	Dendritic polyglycerol sulfate
