# Supplementary material for: Interaction of Lysozyme with Sulfated β-Cyclodextrin: Dissecting Salt and Hydration Contributions
Source: Molecules. 2026 Jan 20;31(2):372. doi: 10.3390/molecules31020372 (PMC12844233; doi:10.3390/molecules31020372)
Supplement: Supplementary file 1 [file molecules-31-00372-s001.zip › molecules-4060764-supplementary.pdf]

Supporting Information

Interaction of Lysozyme with sulfated β-cyclodextrin: dissecting salt and hydration contributions

Jacek J. Walkowiak [1,2,3,4,*]

[1] DWI – Leibniz-Institute for Interactive Materials e.V, Forckenbeckstraße 50, 52074 Aachen, Germany.

[2] Institute of Technical and Macromolecular Chemistry, RWTH Aachen University, Worringerweg 2, 52074 Aachen, Germany.

[3] Aachen-Maastricht Institute for Biobased Materials (AMIBM), Maastricht University, Urmonderbaan 22, 6167 RD Geleen, the Netherlands.

[4] Department of Chemistry, Inorganic Chemistry III, Northern Bavarian NMR Centre, University of Bayreuth, Universitätsstrasse 30, Bayreuth, 95440 Germany.

Correspondence: jacek.walkowiak@uni-bayreuth.de

1. Isothermal Titration Calorimetry

Table S1. Binding parameters for the Lys/β-CDS complex formation as determined by ITC.

c_s (mM)	[β-CDS] (mM)	K_b (M^{-1})	Temp. (K)	ΔG_b (kJ/mol)	ΔH_{ITC} (kJ/mol)	N
20	0.005	1.06E+08	293	-45.0 ± 0.4	-67.0 ± 2.5	0.9
		6.90E+07	296	-44.4 ± 0.5	-62.8 ± 2.5	1
		6.41E+07	300	-44.8 ± 0.8	-60.3 ± 3.9	1
		5.29E+07	303	-44.8 ± 0.5	-61.1 ± 3.8	1
		4.63E+07	307	-45.1 ± 0.5	-64.5 ± 2.1	0.9
		4.03E+07	310	-45.1 ± 0.7	-56.5 ± 3.9	1.1
40	0.005	1.84E+07	293	-40.7 ± 0.4	-65.3 ± 2.1	0.9
		1.53E+07	296	-40.7 ± 0.6	-67.4 ± 3.2	0.9
		1.25E+07	300	-40.8 ± 0.4	-60.3 ± 3.9	0.9
		1.03E+07	303	-40.7 ± 0.1	-58.2 ± 3.8	1
		9.01E+06	307	-40.9 ± 0.4	-57.4 ± 3.8	1
		7.69E+06	310	-40.9 ± 0.5	-54.8 ± 3.9	1
60	0.1	4.33E+06	293	-37.2 ± 0.3	-57.8 ± 4.9	0.9
		3.16E+06	296	-36.8 ± 0.3	-61.1 ± 3.4	0.9
		3.05E+06	300	-37.2 ± 0.4	-55.7 ± 6.1	0.9
		2.76E+06	303	-37.4 ± 0.8	-51.9 ± 5.6	0.9
		2.37E+06	307	-37.5 ± 0.4	-48.6 ± 4.9	0.9
		1.96E+06	310	-37.4 ± 0.6	-42.7 ± 6.5	1
80	0.1	9.52E+05	293	-33.5 ± 0.6	-53.2 ± 4.5	1

8.00E+05	296	-33.5 ± 0.6	-47.7 ± 4.3	0.9
4.63E+05	300	-32.5 ± 0.1	-45.6 ± 6.5	0.9
4.10E+05	303	-32.6 ± 0.1	-42.3 ± 6.8	0.9
4.12E+05	307	-33.0 ± 0.1	-36.0 ± 3.5	0.9
2.37E+04*	307	-25.7 ± 6.2*	- 15.6 ± 8.2*	0.2*
3.53E+05	310	-32.9 ± 0.1	-31.4 ± 3.5	0.9
2.28E+7*	310	-43.7 ± 8.9*	-12.9 ± 8.5*	0.2*

*For all measurements the concentration of lysozyme was constant [Lys] = 1.0 mM. *indicates the binding parameters obtained for the second binding site as a result of TSIS model application.*

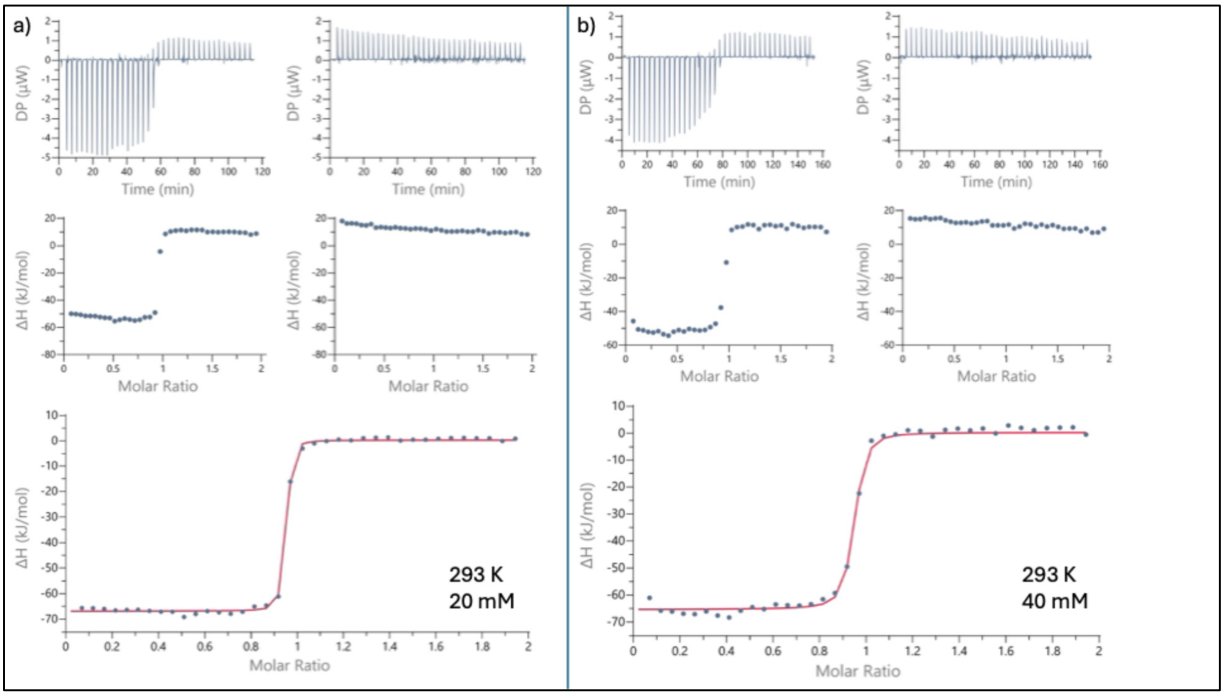

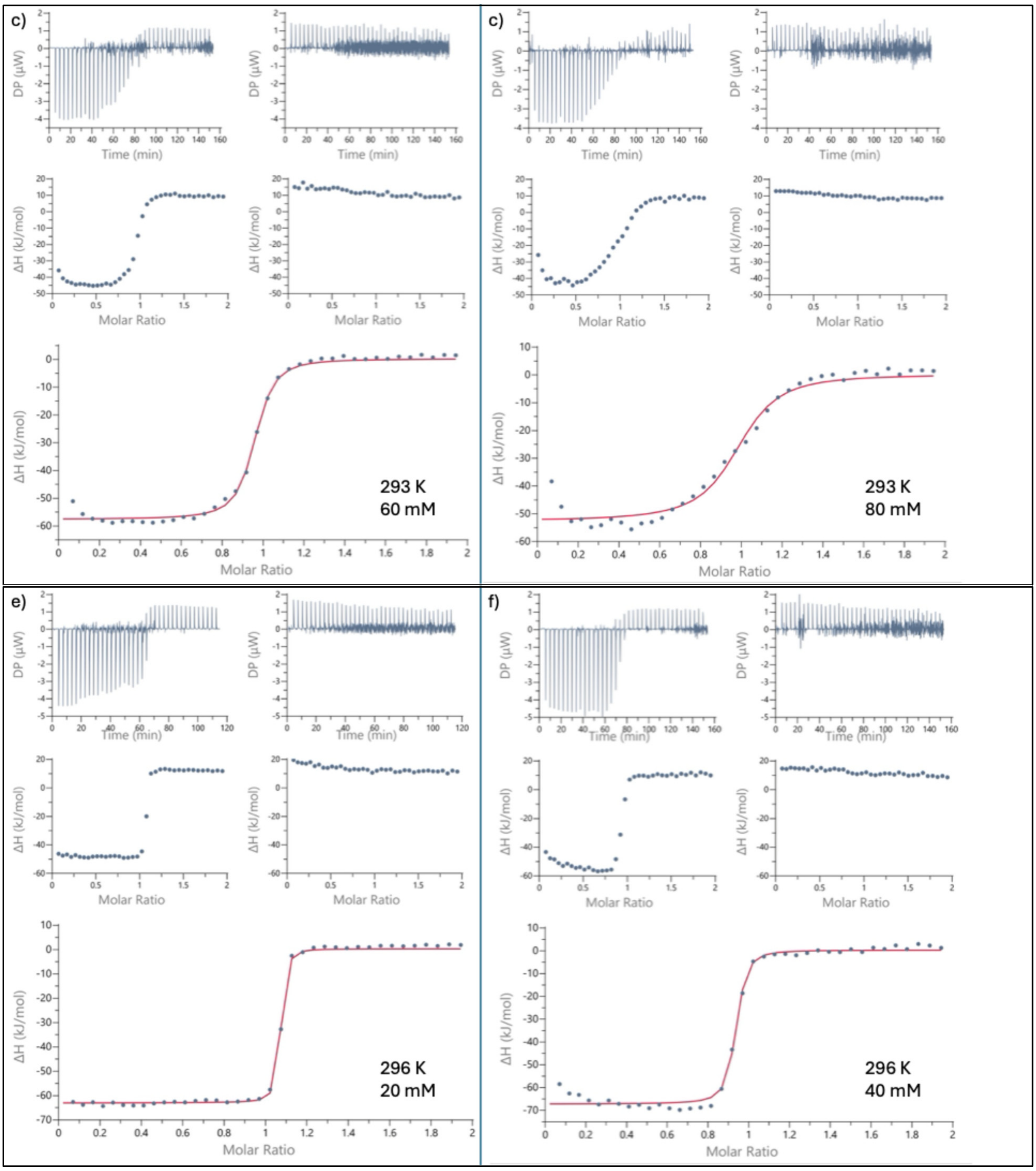
c)
DP (μW)
Time (min)
ΔH (kJ/mol)
Molar Ratio
293 K
60 mM
c)
293 K
80 mM
e)
296 K
20 mM
f)
296 K
40 mM

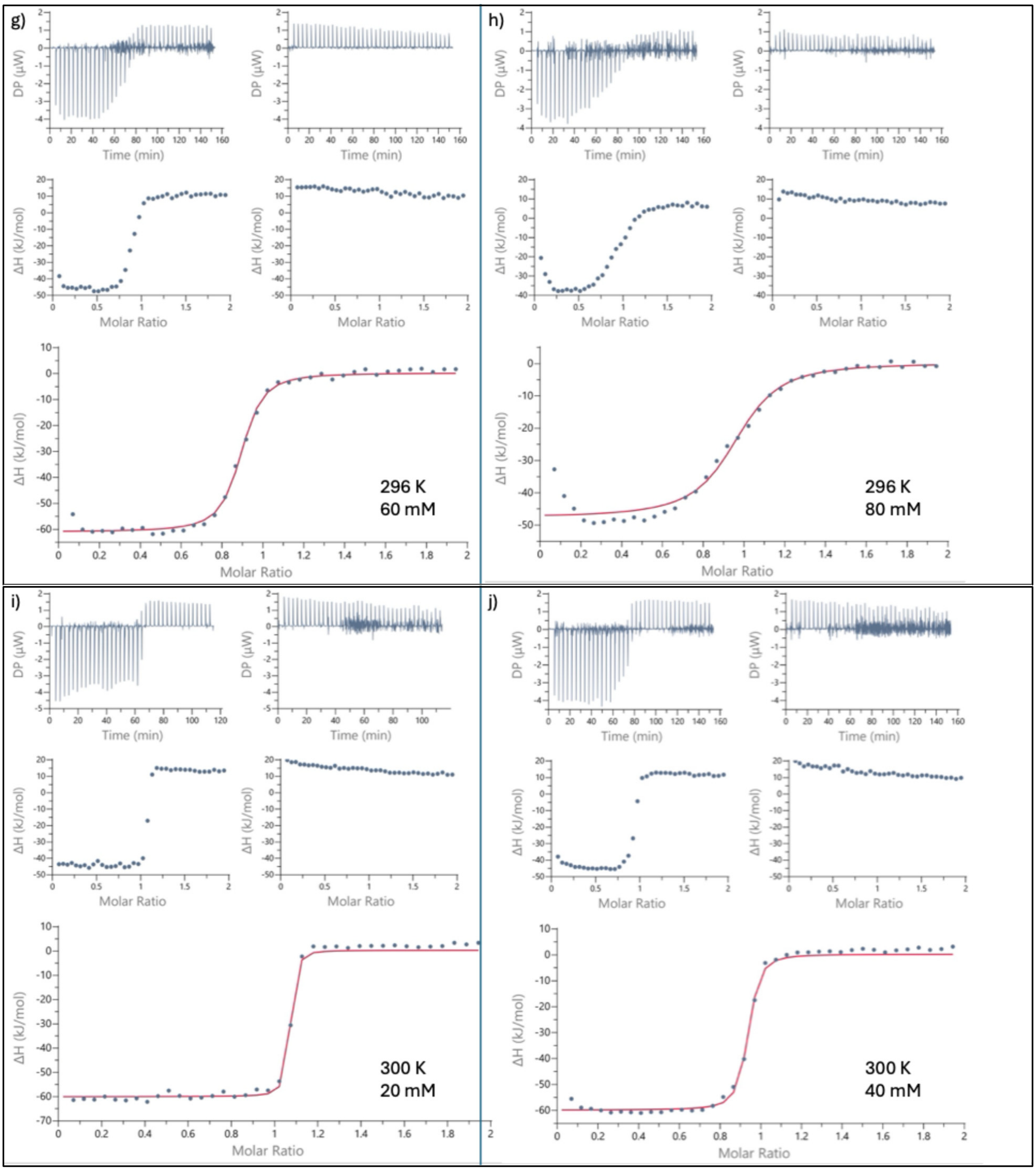

g)
DP (μW)
Time (min)
ΔH (kJ/mol)
Molar Ratio
296 K
60 mM
h)
296 K
80 mM
i)
300 K
20 mM
j)
300 K
40 mM

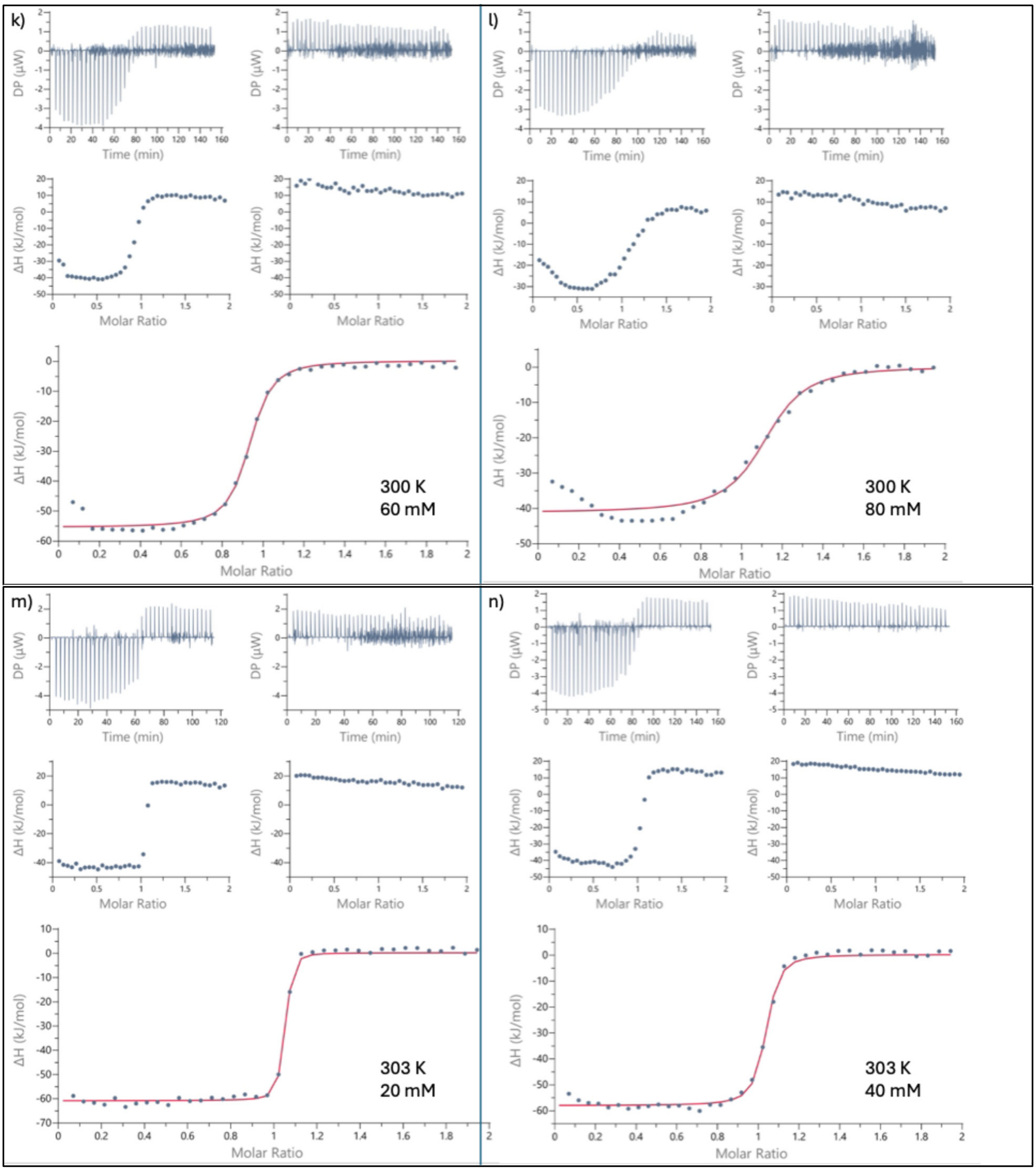
k)
l)
m)
n)
DP (μW)
Time (min)
ΔH (kJ/mol)
Molar Ratio
300 K
60 mM
300 K
80 mM
303 K
20 mM
303 K
40 mM

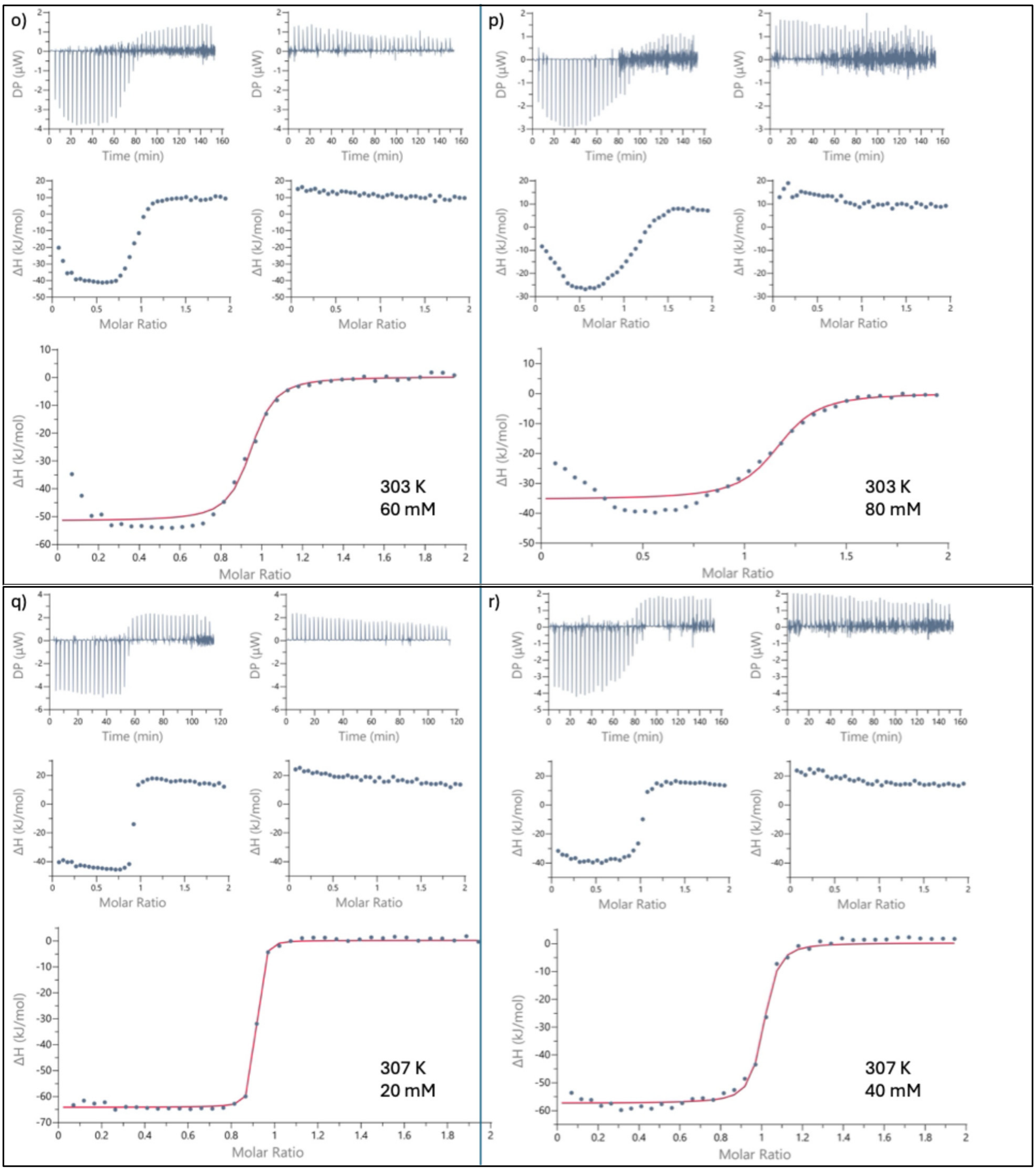
o)
p)
q)
r)
DP (μW)
Time (min)
ΔH (kJ/mol)
Molar Ratio
303 K
60 mM
303 K
80 mM
307 K
20 mM
307 K
40 mM

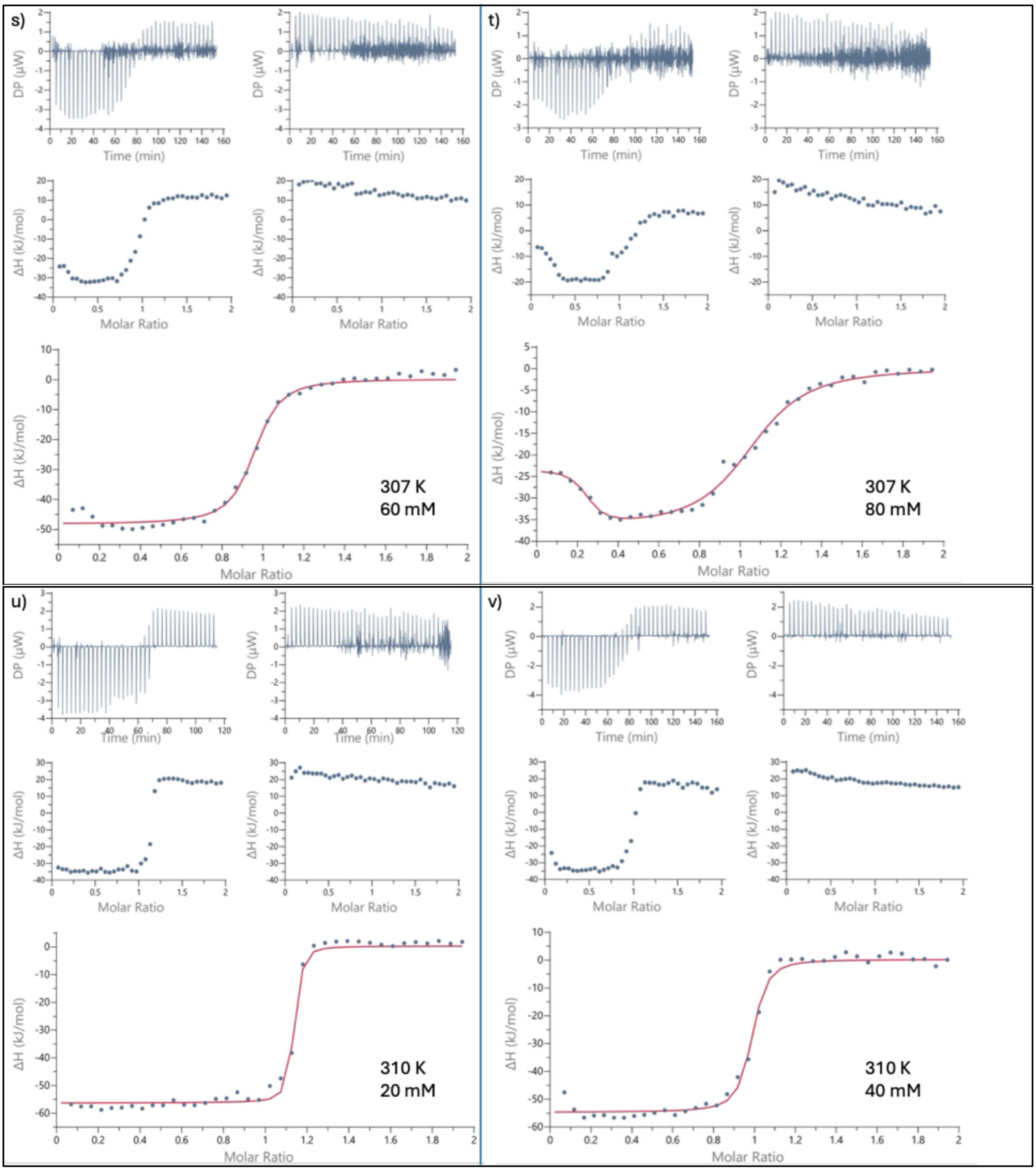

s)
307 K
60 mM
t)
307 K
80 mM
u)
310 K
20 mM
v)
310 K
40 mM
DP (μW)
Time (min)
ΔH (kJ/mol)
Molar Ratio

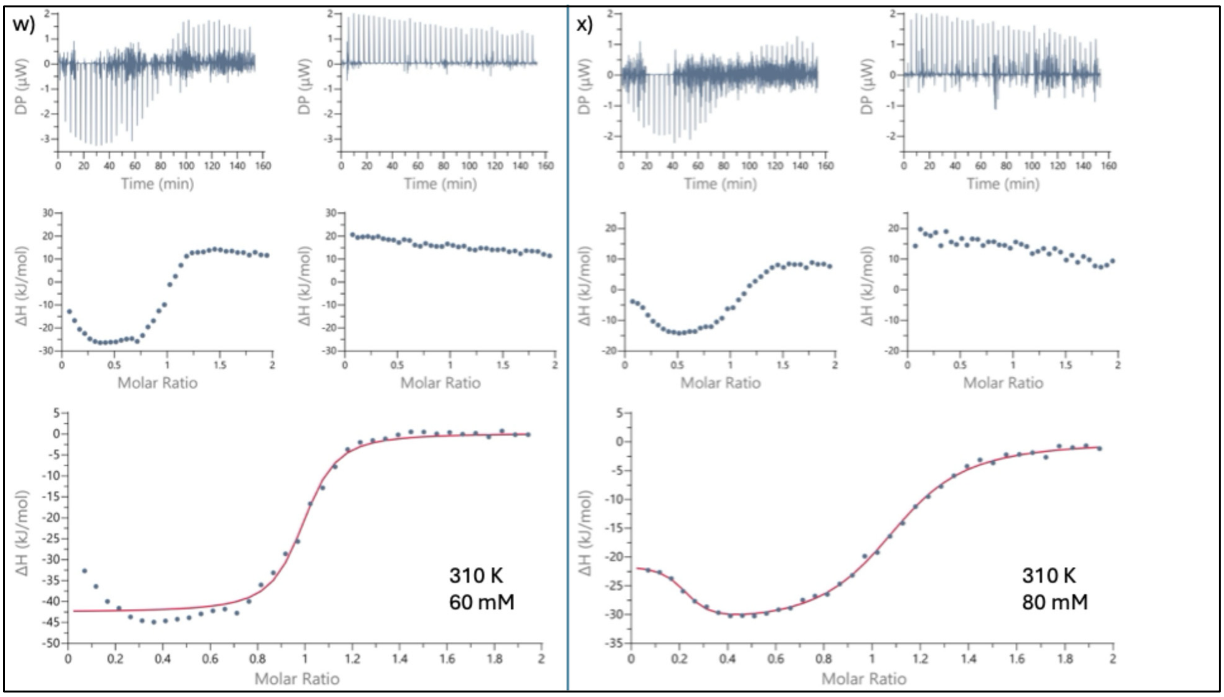

Figure S1. ITC data of the adsorption of β-CDS to Lys (top left) and corresponding data of the Lys heat of dilution (top right). The bottom panels show integrated heats of adsorption of β-CDS to Lys. Solid red line presents the SSIS or TSIS (t and x) fit. Molar Ratio always display the number of Lys molecules per number of β-CDS molecules.

2. Evaluation of ITC Data

2.1.Single Set of Identical Binding Sites (SSIS) Model:

The single set of independent binding site (SSIS) model is based on the Langmuir equation.[1] It assumes equilibrium between the unoccupied binding sites within the macromolecule, the number of protein molecules in solution and the macromolecule occupied binding sites. In principle it relates the fraction of adsorption sites in macromolecule containing bound protein molecules θ to the binding constant K_b:

$$\theta = \frac{K_b[P]}{1+K_b[P]} \tag{S1}$$

where *[P]* is the concentration of free protein molecules in solution. Since the total concentration of $[P]_t$ in the solution is known, *[P]* is connected to the $[P]_t$ as follows:

$$[P]_t = [P] + N\theta[M] \tag{S2}$$

For macromolecule containing *N* adsorption sites, θ is N_b/N where N_b represents the number of protein molecules bound per macromolecule and *[M]* is the total macromolecule concentration in solution. Subtracting equation (S1) into equation (S2) gives:

$$[P]_t = [P] + \frac{NK_b[P][M]}{1+K_b[P]} \tag{S3}$$

Solving of equation (S1) for *[P]* leads to a quadratic equation:

$$\theta^2 - \theta\left[1 + \frac{[P]_t}{N[M]} + \frac{1}{NK_b[M]}\right] = 0 \quad \text{(S4)}$$

The heat Q after each injection i is equal to:

$$Q = [M]V_0 N\theta\Delta H^{ITC} \quad \text{(S5)}$$

Solving the equation (S4) for θ and then substituting this into equation (S5) gives:

$$Q = \frac{N[M]\Delta H^{ITC}V_0}{2}\left[1 + \frac{[P]_t}{N[M]} + \frac{1}{NK_b} - \sqrt{\left(1 + \frac{[P]_t}{N[M]} + \frac{1}{NK_b[M]}\right)^2 - \frac{4[P]_t}{N[M]}}\right] \quad \text{(S6)}$$

The analysis includes the effect of the increase of the volume during titration. The experimental data are fitted by calculating the heat change of the solution ΔQ_i released with each injection i and corrected for displaced volume ΔV_i

$$\Delta Q_i = Q_i + \frac{dV_i}{V_0}\left[\frac{Q_i + Q_{i-1}}{2}\right] - Q_{i-1} \quad \text{(S7)}$$

The process of fitting experimental data involves initial guesses for N, K_b and ΔH^{ITC}; calculation of ΔQ_i for each injection and comparison of these values with the measured heat for the corresponding experimental injections; improvement in the initial values based on the Marquardt methods. The iteration of the above procedure proceeds until the satisfactory fit is achieved.[2]

2.2.Two Sets of Independent Binding Sites (TSIS) Model

This model represents the binding process in which the macromolecule has two non-identical binding sites. Each set of binding sites is characterized by a binding constants K_{b1} and K_{b2} describing the binding affinity of a ligand to the corresponding site. There are six free parameters involved in this model: The binding constants K_{b1} and K_{b2}, the molar heat of binding ΔH^{1ITC} and ΔH^{2ITC}, and respective number of binding sites N_1 and N_2. Each type of site is therefore characterized by its own fractional saturation θ_1 and θ_2. Knowing the total concentrations of protein $[P]_t$ and the macromolecule $[M]_t$ in the solution, the unknown free protein concentration *[P]* can be related by the following equation:

$$[P]_t = [P] + [M]_t(N_1\theta_1 + N_2\theta_2) \quad \text{(S8)}$$

Solving equation (S1) for $\theta 1$ and $\theta 2$ and substituting into equation (S8) gives:

$$[P]_t = [P] + \frac{N_1[M]_t[P]K_{b1}}{1+[P]K_{b1}} + \frac{N_2[M]_t[P]K_{b2}}{1+[P]K_{b2}} \quad \text{(S9)}$$

Solving the equation (S9) for *[P]* results in a cubic equation of the form:

$$[P]^3 + p[P]^2 + q[P] + r = 0 \quad \text{(S10)}$$

where:

$$p = \frac{1}{K_{b1}} + \frac{1}{K_{b2}} + (N_1 + N_2)[M]_t - [P]_t \tag{S11}$$

$$q = \left(\frac{N_1}{K_{b2}} + \frac{N_2}{K_{b2}}\right)[M]_t - \left(\frac{1}{K_{b1}} + \frac{1}{K_{b2}}\right)[P]_t + \frac{1}{K_{b1}K_{b2}} \tag{S12}$$

$$r = \frac{-[P]_t}{K_{b1}K_{b2}} \tag{S13}$$

Equations (S9) and (S10) are solved numerically for *[P]* in the calorimetric software using Newton's method once the fitting parameters N_1, N_2, K_{b1}, K_{b2} and the bulk concentrations are assigned. The values for θ_1 and θ_2 are then given by substitution of *[P]* into equation (S1).
After each injection, the heat Q of the solution within the volume V_0 of the calorimetric cell is equal to:[2]

$$Q = [M]_t V_0 (N_1 \theta_1 \Delta H_1^{ITC} + N_2 \theta_2 \Delta H_2^{ITC} \tag{S14}$$

The experimental data are fitted by calculating the heat change of the solution ΔQ_i released with each injection i and corrected for the displaced volume ΔV_i.

3. Matlab script used to fit binding parameters to equation 3:

```
% Used data values:
T = [293, 296, 300, 303, 307, 310, 293, 296, 300, 303, 307, 310, 293, 296, 300, 303, 307, 310, 293, 296,
300, 303, 307, 310]'; % [K]
cs = [0.02, 0.02, 0.02, 0.02, 0.02, 0.02, 0.04, 0.04, 0.04, 0.04, 0.04, 0.04, 0.06, 0.06, 0.06, 0.06, 0.06,
0.06, 0.08, 0.08, 0.08, 0.08, 0.08, 0.08]'; % [M]
dG = [-45.0, -44.4, -44.8, -44.8, -45.1, -45.1, -40.7, -40.7, -40.8, -40.7, -40.9, -40.9, -37.2, -36.8, -37.2, -
37.4, -37.5, -37.3, -33.5, -33.4, -32.5, -32.6, -33.0, -32.9]'; % [kJ/mol]
dGerr = [0.4, 0.5, 0.8, 0.5, 0.5, 0.7, 0.4, 0.6, 0.4, 0.1, 0.4, 0.5, 0.3, 0.3, 0.4, 0.8, 0.4, 0.6, 0.6, 0.6, 0.1, 0.1,
0.1, 0.1]'; % [kJ/mol], errors


R = 0.008314; % [kJ/(mol*K)]


% Model function: g(params, T, cs)
% params = [Δnci, ΔH0, ΔS0, dΔcp_dcs, T0]
g = @(params, T, cs) ...
    R*T.*params(1).*log(cs) + ...
    params(2) - T*params(3) + ...
    params(4)*cs.*(T - params(5) - T.*log(T./params(5)));


% Initial parameter guess: [Δnci, ΔH0, ΔS0, dΔcp_dcs, T0]
x0 = [-2, -5, 0.05, -1, 350];


% No bounds
```

```
objfun = @(params) (g(params, T, cs) - dG) ./ dGerr;


opts = optimoptions('lsqnonlin', 'Display', 'off');
[param_fit, resnorm] = lsqnonlin(objfun, x0, [], [], opts);


% Display fitted parameters
Deltanci      = param_fit(1)
Deltah0       = param_fit(2)
Deltas0       = param_fit(3)
dDeltacp_dcs  = param_fit(4)
T0            = param_fit(5)


% Plot ΔGb vs salt concentration, log x-axis, at selected temperatures
figure;
hold on;
colors = lines(numel(unique(T)));
csvals = logspace(log10(min(cs)), log10(max(cs)), 100)';
Ti = unique(T);
for idx = 1:numel(Ti)
    dG_line = g(param_fit, Ti(idx)*ones(size(csvals)), csvals);
    plot(csvals, dG_line, '-', 'Color', colors(idx,:), 'LineWidth', 2);
end
errorbar(cs, dG, dGerr, 'ko', 'MarkerFaceColor', 'k', 'LineStyle', 'none', 'CapSize', 6, 'LineWidth', 1);
set(gca, 'xscale', 'log');
xlabel('Salt concentration, c_s [M]');
ylabel('Free energy of binding, \DeltaG_b [kJ/mol]');
legend(arrayfun(@(t) sprintf('T = %d K', t), Ti, 'UniformOutput', false), 'Location','best');
title('\DeltaG_b vs salt concentration (log scale) at different T');
grid on;


% Plot ΔGb vs temperature at selected salt concentrations (linear x-axis)
figure;
hold on;
csi = unique(cs);
colors = lines(numel(csi));
Tvals = linspace(min(T), max(T), 100)';
for idx = 1:numel(csi)
    dG_line = g(param_fit, Tvals, csi(idx)*ones(size(Tvals)));
    plot(Tvals, dG_line, '-', 'Color', colors(idx,:), 'LineWidth', 2);
end
errorbar(T, dG, dGerr, 'ko', 'MarkerFaceColor', 'k', 'LineStyle', 'none', 'CapSize', 6, 'LineWidth', 1);
xlabel('Temperature, T [K]');
ylabel('Free energy of binding, \DeltaG_b [kJ/mol]');
legend(arrayfun(@(c) sprintf('c_s = %.3f M', c), csi, 'UniformOutput', false), 'Location','best');
```

```
title('\DeltaG_b vs temperature at different salt concentrations');
grid on;
```

References

1. Indyk, L.; Fisher, H.F. [17] Theoretical Aspects of Isothermal Titration Calorimetry. In *Methods in Enzymology*; 1998; pp. 350–364.
2. Lin, L.N.; Mason, A.B.; Woodworth, R.C.; Brandts, J.F. Calorimetric Studies of the Binding of Ferric Ions to Human Serum Transferrin. *Biochemistry* 1993, *32*, 9398–9406, doi:10.1021/bi00087a019.
